# Modeling protein–nucleic acid complexes with extremely large conformational changes using Flex-LZerD

**DOI:** 10.1002/pmic.202200322

**Published:** 2022-12-25

**Authors:** Charles Christoffer, Daisuke Kihara

**Affiliations:** 1 Department of Computer Science, Purdue University, West Lafayette, Indiana, USA; 2 Department of Biological Sciences, Purdue University, West Lafayette, Indiana, USA; 3 Purdue University Center for Cancer Research, Purdue University, West Lafayette, Indiana, USA

**Keywords:** flexible assembly, flexible docking, nucleic acid docking, protein structure prediction, protein–nucleic acid docking

## Abstract

Proteins and nucleic acids are key components in many processes in living cells, and interactions between proteins and nucleic acids are often crucial pathway components. In many cases, large flexibility of proteins as they interact with nucleic acids is key to their function. To understand the mechanisms of these processes, it is necessary to consider the 3D atomic structures of such protein–nucleic acid complexes. When such structures are not yet experimentally determined, protein docking can be used to computationally generate useful structure models. However, such docking has long had the limitation that the consideration of flexibility is usually limited to small movements or to small structures. We previously developed a method of flexible protein docking which could model ordered proteins which undergo large-scale conformational changes, which we also showed was compatible with nucleic acids. Here, we elaborate on the ability of that pipeline, Flex-LZerD, to model specifically interactions between proteins and nucleic acids, and demonstrate that Flex-LZerD can model more interactions and types of conformational change than previously shown.

## 1 | INTRODUCTION

Protein–nucleic acid interactions are a core part of many biological processes, playing roles in transcription, its regulation, and more [1]. To understand the mechanisms of these processes at a molecular level, the 3D structures of the complexes involved are crucial. While protein–nucleic acid complex structures determined by experiment are being accumulated in the Protein Data Bank (PDB) [2], experiments are slow and expensive. Moreover, structures of heterogeneous complexes are often extremely difficult to determine experimentally. Thus, when a complex structure has not yet been experimentally determined, computational tools can be used to construct coordinate models [3]. A so-called protein docking program can take component proteins, called subunits, as input and assemble them into coordinate models of the full complex. Many general protein–protein docking methods and specialized versions thereof have been developed, such as ZDOCK [4], HADDOCK [5], ClusPro [6], RosettaDock [7], HEX [8], SwarmDock [9], and ATTRACT [10]. Even protein structure prediction methods like AlphaFold [11] have been reworked to be able to output multimeric structures [12], although neither regular AlphaFold nor AlphaFold-Multimer function when nucleic acids are involved. The rigid-body docking method LZerD [13–16] has ranked highly in the server category in recent rounds of CAPRI [17, 18], the blind communitywide assessment of protein docking methods, and has been shown able to sample protein–nucleic acid interaction poses [19]. Other past rigid body methods such as HDOCK [20] and NPdock [21] have also been developed, specifically with nucleic acids in mind.

One major complication in computational complex modeling is the flexibility of macromolecules in general. Even with state-of-the-art conformational sampling techniques, existing docking methods struggle to handle substantial protein conformational changes beyond roughly 2 Å root-mean-square deviation (RMSD) [22–24]. Extreme conformational changes on the order of 10 Å RMSD and above, though well above 2 Å RMSD and thus constituting difficult targets, are quite common, and are often related to protein function [25–31]. These cases include for example rearrangements or reorientations between some domains of the flexible protein along with any changes to other domains or regions otherwise separating them. For example, transcription factor IIB (TFIIB) undergoes such a conformational change when it binds to DNA and facilitates transcription initiation. When a TATA-box-binding protein binds to DNA, the DNA is distorted and stably held in a conformation to which TFIIB can bind through a combination of a larger rearrangement of its cyclin-like domains about a linker and a much smaller backbone deformation internal to the domains. This conformational change enables the cyclin-like domains of TFIIB to interact simultaneously and differentially with the major and minor grooves upstream and downstream of the TATA-box, thus nucleating a functional preinitiation complex with the proper directionality along the DNA [32]. Signal recognition particles have a protein component which takes on different conformations as it interacts with RNA during a cycle of cotranslational protein targeting [33]. Antibodies can even be designed to target nucleic acids, entailing large conformational changes [34]. Techniques capable of modeling such extreme protein conformational changes related to nucleic acid binding thus have the potential to elucidate many cellular processes in many cellular contexts.

Algorithms have been developed which predict the directions or degrees of conformational change a given protein might undergo [35–38], including as part of complex formation [39]. Experimentalists often observe far more drastic conformational changes [25–31] than those with a few angstroms RMSD difference which older assembly techniques struggle with or are unable to handle [22]. There are many ways to approach such small flexibility. The soft surface representation of LZerD [13, 14] can tolerate differences in side chains, for example. When the protein backbone must be moved, it can be sampled explicitly by many techniques including by normal modes [24, 40], by Monte Carlo simulation [41, 42], or by molecular dynamics [43, 44]. Docking with explicit sampling methods can require cross-docking, necessitating precise sampling or extraordinarily fast docking to maintain reasonable running times [45, 46]. Despite substantial advancements, more classical protein docking methods cannot generally model large-scale conformational changes of ordered ligand proteins. Older methods can handle some lesser flexibility [24], but cannot seem to break a barrier at larger RMSDs of conformational change.

In our previous work, we targeted the regime of ≥10.0 Å RMSD coherent flexibility, and developed a new method called Flex-LZerD. Flex-LZerD is based on the observation that often the formation of complexes involving the flexibility discussed above involves interactions of a small number of almost rigid domains of the ligand protein with the receptor. Following this principle, Flex-LZerD constructs complex models by docking domains, which are extracted from a ligand structure, independently of each other. An iterative fitting procedure based on normal mode analysis and energy minimization then docks the entire ligand structure, including residues not part of the extracted domains, to the receptor. In that work, we tested Flex-LZerD mainly on protein–protein complexes and also applied it to protein–nucleic acid complexes. Here, we focus on predicting structures of protein–nucleic acid complexes with Flex-LZerD. We applied Flex-LZerD to a wider class of protein–nucleic acid interactions than the previous work, including for example transcription factors, RNA-targeting antibodies, and ribonucleases, for a total of nine new targets. Flex-LZerD modeled the protein–nucleic acid interfaces to within 6.0 Å RMSD for five out of the nine (55.6%) added cases and 11 out of 17 (64.7%) overall, which include protein–nucleic acid targets from the previous work. Using standard CAPRI criteria for docking evaluation [47], Flex-LZerD modeled six out of the nine added cases (66.7%) correctly, and 14 out of 17 (82.4%) overall. Additionally, Flex-LZerD demonstrated the capacity to sample correct poses, even when it cannot select them. The Flex-LZerD flexible fitting code is available from https://github.com/kiharalab/Flex-LZerD.

## 2 | MATERIALS AND METHODS

### 2.1 | Protein–nucleic acid complex dataset construction

The dataset used in this work was constructed by scanning the PDB using all-vs-all BLAST [48] for pairs of protein–nucleic acid complex entries (8479 entries) containing corresponding subunits with at least 90% sequence alignment coverage and 10.0 Å RMSD of conformational difference (1590 pairs), 70% sequence identity (1350 pairs). Pairs excluding exact PDB entries from the original Flex-LZerD paper [19] were grouped by single-linkage clustering (21 clusters) to direct the manual inspection. Pairs were then filtered by manual inspection to only include targets with large-scale conformational changes in subunits with an interface with a nucleic acid. This procedure finally yielded nine protein–nucleic acid complex targets. The eight protein–nucleic acid targets from the previous work [19] were then added, for a total dataset of 17 targets. The dataset is detailed in Table 1, with targets used in the previous work indicated with asterisks. The combined dataset has two complexes that are in the same protein family, one from the previous work and the other that was newly added. They are DNA polymerase IV (2W9B and 2IMW) and elongation factor Tu (1OB2 and 1TTT). The sequence identities of the two entries are shown in the caption of Table 1. The docking results of these entries are individually discussed.

### 2.2 | Overview of Flex-LZerD

**FIGURE 1:**
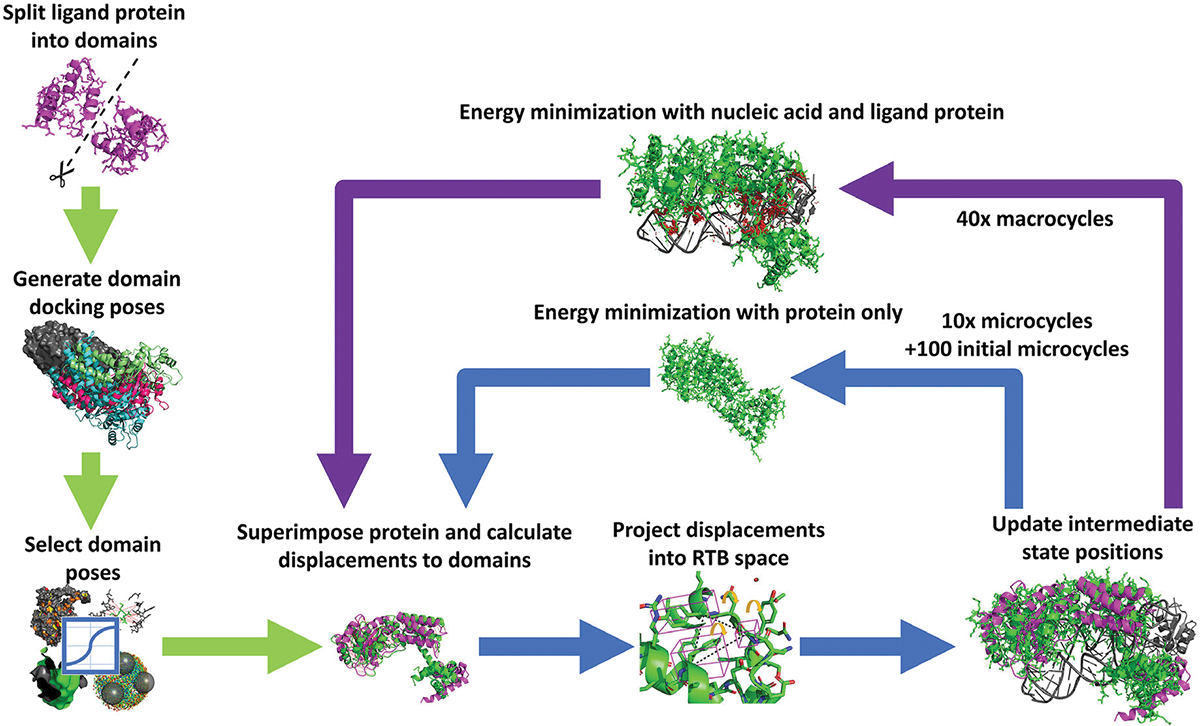
Overall flow of the Flex-LZerD method. Green: the initial stage of the pipeline, which here yields partially assembled protein–nucleic acid complex models. Two domains are extracted from the protein ligand, the domains are docked to the receptor, and domain poses are selected via a consensus scoring function. Blue: the microcycle loop of the pipeline, where atom coordinates are updated according to normal mode displacements and energy minimization of the ligand. The energy minimization using Phenix takes into account bonds and clashes, but not long-range interactions or any dynamics terms. Purple: the macrocycle loop of the pipeline, where atom coordinates are updated according to an otherwise identical energy minimization of the ligand in the presence of the receptor.

Flex-LZerD is designed around the observation that the formation of complexes involving large-scale collective motion often involve little conformal change within individual domains, at least relative to the magnitude of the whole-protein conformational change. The protocol then assumes that a target complex involves interactions of a small number of nearly rigid domains of the ligand protein with the receptor, which can be another protein or a nucleic acid. The overall flow, shown in Figure 1, is thus as follows. After two domains have been expertly extracted from an unbound input protein structure, the domains are assembled with the receptor structure independently of each other using rigid-body docking. For each domain, 100 top-scoring poses are selected. Each combination taking one pose from each domain is then input, along with the full unbound ligand structure, to an iterative elastic network-based fitting procedure that then assembles the entire full-atom complex structure, including residues not part of the extracted domains. The 10 top-scored models are then considered as the output of the Flex-LZerD pipeline. Below, we describe more details at each step of the pipeline. For further details, see the original paper [19].

### 2.3 | Partial assembly with ligand domains

Domain models were generated for each ligand by identifying structural domains and removing other residues, with no limit on the sequence distance that can separate the domains. Each ligand protein structure domain was then docked with the receptor structure using LZerD [13, 49], a shape complementarity-based rigid-body docking algorithm which is tolerant to some small conformational change via a soft surface representation. LZerD uses geometric hashing to rapidly generate many docking poses, which are then scored according to their surface shape complementarity. The set of docked models generated by LZerD for each domain was ordered by the LZerD shape score and truncated to 50,000 models. Each set was then clustered with a ligand pose RMSD cutoff of 4.0 Å to remove redundant poses. The cutoff of 4.0 Å was chosen here for consistency with past developments and analysis of LZerD which have demonstrated its suitability, including blind prediction in CAPRI [14, 50, 51], intrinsically disordered protein docking [52], and multimeric docking [53].

### 2.4 | Domain and model scoring

To select docked domain poses, Flex-LZerD uses a logistic regression scoring function that combines the knowledge-based scoring functions GOAP [54], DFIRE [55], and ITScorePro [56], which are usually combined into the ranksum score used in LZerD docking [14, 50, 51]. Flex-LZerD additionally includes the LZerD shape score, the cluster size from the usual 4.0 Å RMSD rigid-body docking model clustering, binding site consensus (BSC) terms representing the consensus of residue interaction among the scored models, and order statistic terms highlighting extreme values among the other scoring terms for each model.

The LZerD shape scores and the cluster sizes are taken directly from the initial rigid docking stage. The $BSC_{x,y}$ terms quantify the frequency of residue–residue interactions observed among the generated decoys. There are six BSC terms, calculated from all combinations of two interaction distance cutoffs (5.0 and 10.0 Å) indicated in the y subscript and three sets of interface residues considered (receptor, ligand, and both) indicated in the x subscript. $BSC_{x,y}$ is then calculated for a model by assigning to each residue in x an occupancy, the number of times it is observed in an interacting pose under the cutoff y for the entire space of searched poses, and then summing this occupancy value over the interactions, determined again by the cutoff y, observed in the specific model. The order statistic terms consider if any of the individual scores strongly favor some particular model, even if that model is not ranked highly by consensus of the other scores; they are calculated by standardizing all the component terms into z-scores (centering on the mean and dividing by the standard deviation) and selecting the first, second, and third lowest. Flex-LZerD then combines the component scores in a logistic regression model, which is used to score the model pool. This same scoring function is used to score the final output models of the pipeline as well, carrying over the BSC rather than recalculating. This combined scoring function [52] considering knowledge-based scoring functions from ranksum [14, 50, 51], which have performed well in CAPRI [17, 18, 47, 57, 58], and model consensus features is thus also used to select the top 100 docked poses for each domain.

### 2.5 | Anisotropic network model

Flex-LZerD uses an anisotropic network model (ANM) [59–61] to deform the ligand structure to match the docked domains. In an ANM, atoms are considered as point masses with a simple harmonic spring potential constructed by considering initial distances between atoms, here with a 15.0 Å connectivity cutoff. Principal components of any possible large-scale motions can then be extracted from this potential. Thus, for each pair of atoms i and j among the total n atoms, we build up a harmonic potential U pairwise, adding together all harmonic terms $U_{ij}\approx {∥s_{ij}-s_{ij}^{0}∥}^{2}$, where $s_{ij}$ is the current distance between i and $j,\;s_{ij}^{0}$ is the initial distance between i and j, and constant factors are elided.

To extract components, we calculate the Hessian H of the potential and formulate the eigenproblem $H\vec{s}=\lambda \vec{s}$. The eigenvectors corresponding to the smallest nonzero eigenvalues here are then the normal modes of the system which allow representation of a short segment of a large-scale conformational change. The first 20 modes are used by Flex-LZerD. Flex-LZerD further uses the rotations and translations of blocks (RTB) [62] projection method, here implemented in the ProDy framework [63], which facilitates construction of a much smaller approximate Hessian matrix which is much faster to diagonalize. This reduced diagonalization is then used to calculate full-atom modes.

### 2.6 | Iterative fitting to docked domains

After domain poses have been selected, all poses for each domain are considered combinatorially pairwise and flexibly fit them to dock against the receptor via iterated normal mode analysis and energy minimization, resulting in 100 × 100 = 10,000 output models. For a given pair, the full unbound ligand structure is superimposed to minimize the RMSD to the domain pose pair, corresponding to the superimposition step in Figure 1. Modes are then calculated in a reduced representation as described above. Using the modes, the coordinates of the ligand atoms are displaced in straight lines to update the fitted state, corresponding to the projection and update step in Figure 1. To circumvent the straight-line nature of normal modes, Flex-LZerD applies only a small-amplitude motion at a time, recalculating the Hessian and normal modes and performing a short minimization each iteration to avoid stereochemical violations as illustrated in Figure 1. The motion is selected by directly projecting displacements from the ligand to the docked domains into the normal mode subspace. The projection can be obtained by taking a simple dot product with each eigenvector; the truncated modes still span a linear space and are an orthogonal basis for it. This procedure has the advantage that since there is a normal mode component for each atom in the ligand, ligand atoms not modeled in the domains can still be updated. The short minimization during each iteration is run using PHENIX [64], and is applied always to the ligand, but also to the receptor during every 10th iteration after the 100th. The fitting continues for 500 iterations or 4 h, whichever is shorter.

## 3 | RESULTS AND DISCUSSION

Flex-LZerD was originally benchmarked on a set of protein complex targets [19] including eight protein–nucleic acid complexes. In that work, Flex-LZerD was shown to model 100% of the nucleic acid targets examined acceptably, where acceptable quality was determined according to the longstanding CAPRI criteria [47], which combines the measures interface RMSD (I-RMSD), ligand RMSD (L-RMSD), and fraction of native contacts satisfied (*f*_nat_) using the thresholds 4.0 Å, 10.0 Å, and 0.10 to obtain a categorical classification of the quality of a model. To calculate I-RMSD, the interface is defined from the native structure as all residues in either subunit containing at least one heavy atom within 10.0 Å of any atom of the other subunit. The backbone atoms of the native interface are then superimposed to the corresponding atoms in the model, and their RMSD is taken as I-RMSD. *f*_nat_ defines native contacting residues using the same heavy atom criterion as used for the interface, except with a 5.0 Å distance cutoff instead. Each pair of residues in contact between the subunits in the native structure is considered a native contact. The fraction of these native contacts which are also present in the model is taken as *f*_nat_. L-RMSD is calculated by first superimposing the model receptor to the native receptor structure. The RMSD of the model ligand to the native ligand is then taken as L-RMSD, without further superimposition. A model is then considered acceptable under the CAPRI criteria if it has an *f*_nat_ of at least 0.10 and has either an I-RMSD of at most 4.0 Å or an L-RMSD of at most 10.0 Å, or both. In this work, we examined an enlarged dataset with nine more protein–nucleic acid targets and discuss the results with the results on the eight targets from the previous work (Table 1).

The modeling results are summarized in Table 2, and output models are available from https://zenodo.org/record/7412584 [65]. The “Domain level” block details the performance of the protein domain docking stage in isolation, while the “Complex level” block details the performance of the final output Flex-LZerD pipeline in comparison to rigid-body docking and nonblind flexible docking. “Flex-fitting-to-native” details the outcome where the input unbound protein was fitted to the correct positions of the individual domains. The goal of this column is to show that the flexible deformation process, which is the core of Flex-LZerD, works well and yields small I-RMSD values in the ideal scenario when the individual correct domain docking poses are known exactly. From the “Rigid-body LZerD” column, it is clear that rigid-body docking generally does not work where extreme flexibility is involved. The one exception was Ribonuclease E, where the focus of the large-scale change was away from the interaction site; the interface was thus sufficiently correct for rigid assembly to sample a CAPRI-acceptable model. In general, however, large-scale flexibility prevents rigid assembly.

**FIGURE 2:**
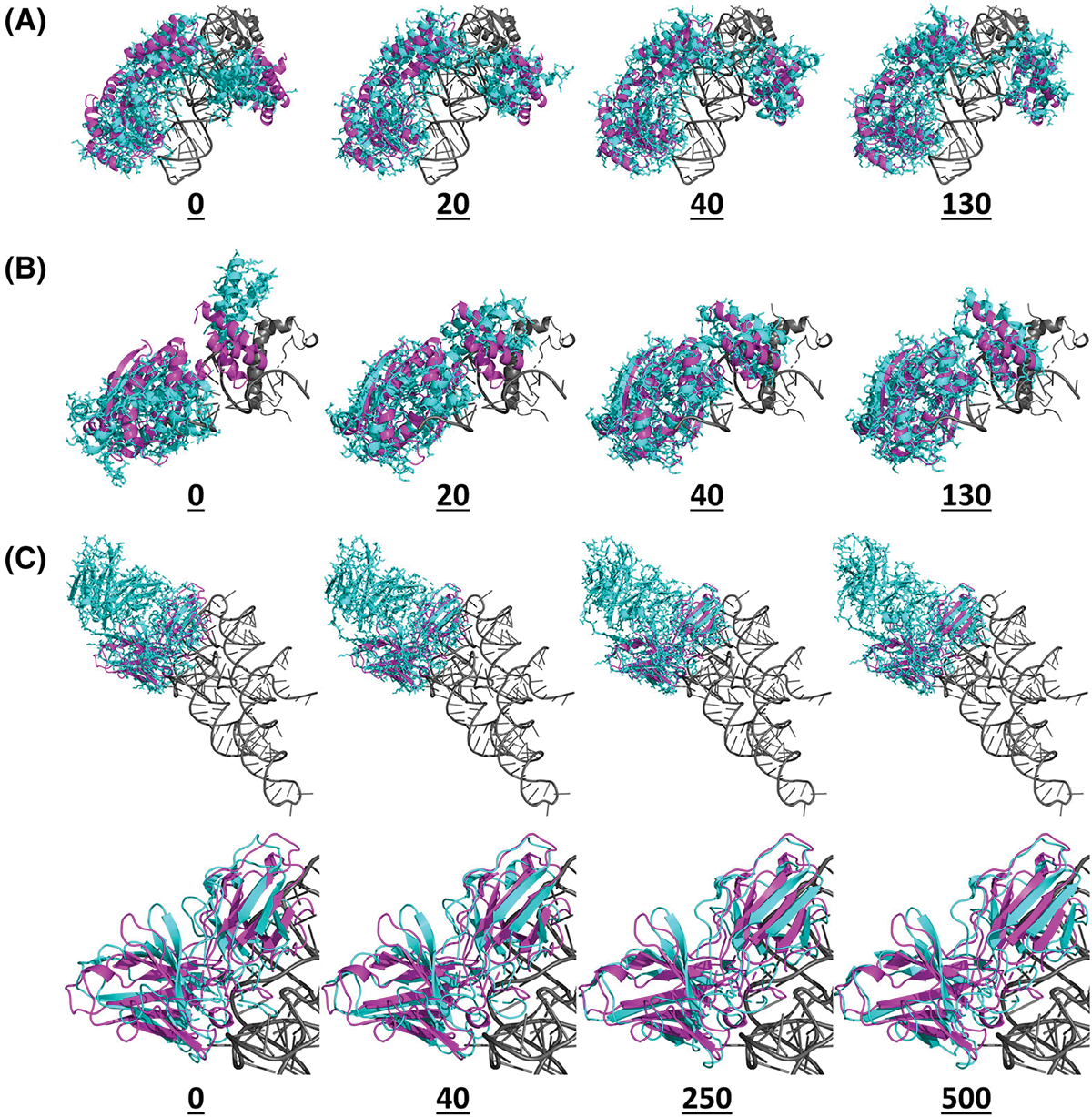
Selections of frames from flexible fitting runs on case study targets. The frame number is indicated below each image. Gray: the receptor structure. Magenta: the docked domain models being fitted to. Cyan: the input unbound ligand protein model being deformed. Frame 0 is the input unbound protein structure superimposed to the docked domain models. (A) SRP54 (PDB 2V3C) is pulled open by the flexible fitting, allowing the model to accept the RNA. The initial frame had an I-RMSD of 10.9 Å, an L-RMSD of 9.5 Å, and an *f*_nat_ of 0.03. Frame 20 had an I-RMSD of 9.6 Å, an L-RMSD of 6.9 Å, and an *f*_nat_ of 0.28. Frame 40 had an I-RMSD of 8.4 Å, an L-RMSD of 5.9 Å, and an *f*_nat_ of 0.31. The final frame of the fitting run shown had an I-RMSD of 6.9 Å, an L-RMSD of 5.5 Å, and an *f*_nat_ of 0.26. (B) ERI1 (PDB 4QOZ) is pulled closed by the flexible fitting, forming a combined interface with the RNA. The initial frame had an I-RMSD of 11.2 Å, an L-RMSD of 15.3 Å, and an *f*_nat_ of 0.07. Frame 20 had an I-RMSD of 6.0 Å, an L-RMSD of 10.9 Å, and an *f*_nat_ of 0.27. Frame 40 had an I-RMSD of 4.7 Å, an L-RMSD of 9.3 Å, and an *f*_nat_ of 0.38. The final frame of the fitting run shown had an I-RMSD of 4.6 Å, an L-RMSD of 8.7 Å, and an *f*_nat_ of 0.40. (C) A Fab (PDB 2R8S) is slowly deformed, gradually deforming the antibody variable region into agreement with the docked domain poses. The top row shows the full complex, while the bottom row shows a magnified view of only the domain regions and the RNA. The initial frame had an I-RMSD of 3.2 Å, an L-RMSD of 20.2 Å, and an *f*_nat_ of 0.44. Frame 40 had an I-RMSD of 3.4 Å, an L-RMSD of 20.1 Å, and an *f*_nat_ of 0.48. Frame 250 had an I-RMSD of 3.7 Å, an L-RMSD of 21.0 Å, and an *f*_nat_ of 0.39. The final frame of the fitting run shown had an I-RMSD of 3.8 Å, an L-RMSD of 21.7 Å, and an *f*_nat_ of 0.33. I-RMSD, interface RMSD; L-RMSD, ligand RMSD; PDB, Protein Data Bank; RMSD, root-mean-square deviation.

In Figure 2, we illustrate how the flexible fitting worked on three cases. The flexible fitting procedure used by Flex-LZerD is able to deform unbound input ligand structures into agreement with docked domain structures. Figure 2A shows SRP54 starting from a closed conformation. It is initially pulled open, and its domains reorient into agreement with the docked domain poses. Finally, it is brought into surface complementarity with the RNA. Figure 2B shows ERI1 starting from an open conformation. It is initially pulled closed, and its domains quickly reorient to match the docked domain poses. Figure 2C shows an RNA-targeting Fab starting from a typical isolated Fab conformation. The domains in this case are not as free to move, and the unbound structure deforms quite slowly to match their independently docked counterparts. Flexible fitting is thus clearly able to fit an unbound structure to a given set of docked domain poses. These targets are further discussed in the case studies below.

On the nine new targets, models of at least acceptable quality were generated in the top 10 selected models for six out of the nine targets (66.7%). On the dataset of 17 targets overall, Flex-LZerD thus correctly modeled 14 of the 17 (82.4%) total targets. Those targets which attained acceptable quality but have I-RMSDs greater than 4.0 Å, as shown in Table 2, have L-RMSDs less than 10.0 Å. The combined scoring function used in Flex-LZerD was also seen here to successfully select acceptable models from the set of 10,000 into the top 10. The best model by I-RMSD in out of all 10,000 (“Best in all” column in Table 2) was on average only 1.3 Å better than the best out of the top 10 (best in top 10 column in Table 2). The only target where an acceptable model was available in the pool, but was not selected, was an RNA-targeting Fab (2R8S). This target is discussed in Section 3.3.

The dataset has two complexes that are in the same protein family, DNA polymerase IV and elongation factor Tu. The two DNA polymerase IV entries, 2W9B and 2IMW, bound to different nucleic acid molecules, which have an RMSD of 5.4 Å. The DNA polymerase IV examined here had a larger conformational change 16.3 versus 18.2 Å (see Table 1 for a complete comparison) but was still modeled to acceptable quality (see Table 2). The other substantially sequence-similar targets, EF-Tu (1TTT) and EF-Tu2 (1OB2, 74% sequence identity), had nearly identical magnitudes of conformational difference between the bound and unbound structures. 1TTT was docked successfully with an I-RMSD of 4.09 Å when top 10 scoring models are considered while the newly added target, 1OB2 had an I-RMSD of 10.21 Å. The newly examined EF-Tu structure (1OB2) is bound to a GDP molecule and contains additional smaller scale conformational changes that the rigid-body domain docking and coarse-grained fitting of Flex-LZerD cannot easily handle. Thus, one domain of EF-Tu was well-docked to the receptor, but the other was not, and the flexible fitting was unable to overcome it. A totally new inclusion was histone H3.3 protein, which binds as part of a tightly wound bundle of protein and DNA. When bound to DNA, a helix of H3.3 moves into the groove of DNA. While the flexible fitting can scale quite well to larger or smaller domains, the rigid-body domain docking however cannot. LZerD was unable to dock the binding helix of H3.3 into the tight binding site.

**FIGURE 3:**
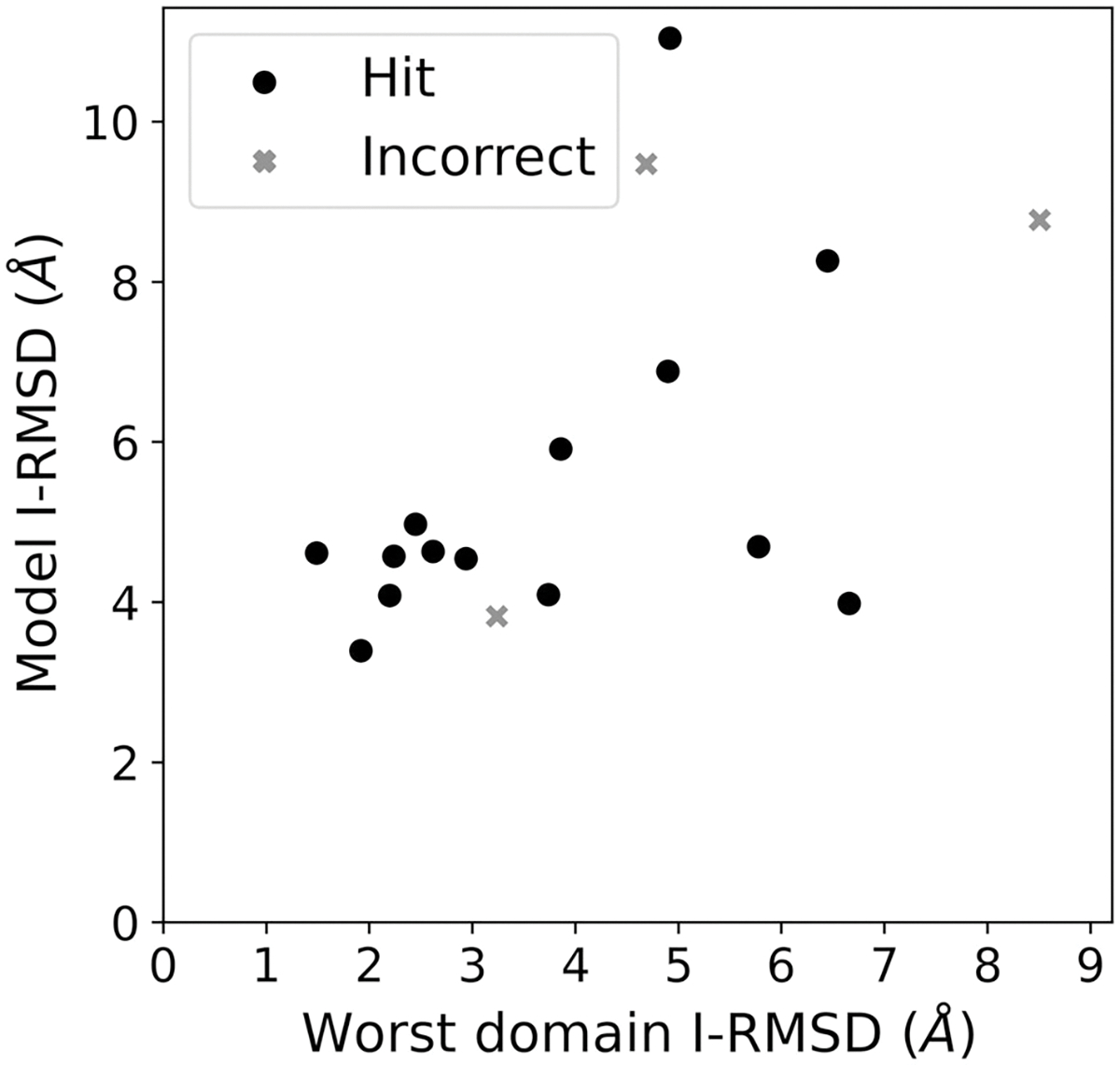
Relationship between quality of docked domain poses and quality of fitted models in terms of I-RMSD. The plot shows I-RMSD for the worst protein domain pose used to build that model versus the I-RMSD for the final complex model that was built. The top hit for each successful target was used. For targets without hits, the model lowest I-RMSD was used. I-RMSD, interface RMSD; RMSD, root-mean-square deviation.

The outcome of modeling with Flex-LZerD naturally depends strongly on the quality of domain pose selection. As can be seen from Table 2, larger deviations in the domain poses without native interactions modeled come with correspondingly large deviations in the full-atom complex models. Figure 3 illustrates this trend of input quality as a function of output quality. All targets where domains were modeled to within 3.0 Å I-RMSD also output final models of acceptable quality. As domain pose I-RMSD increases, so too does fitted model I-RMSD in general.

We discuss two successful cases and one case where Flex-LZerD did not yield a model within 6.0 Å I-RMSD in the top 10.

### 3.1 | Case study 1: SRP54 (PDB 3NDB/2V3C)

**FIGURE 4:**
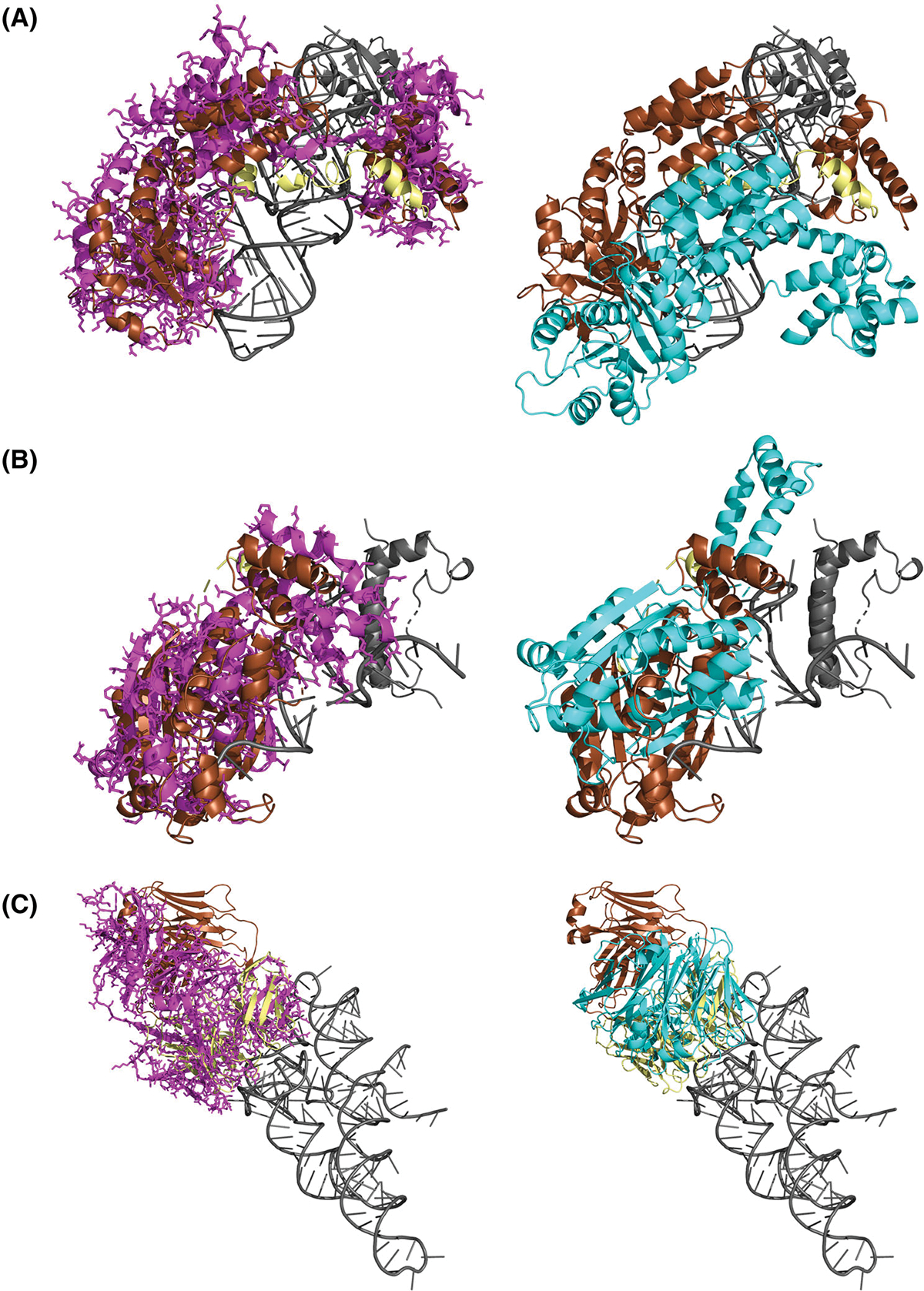
Example modeling of protein–nucleic acid complexes. Gray: the receptor structure. Brown: the potions of the native ligand structure corresponding to the extracted domains used for rigid-body domain docking. Yellow: the potions of the native ligand structure which do not correspond to extracted domains. Magenta (left): the top Flex-LZerD model output, except for panel (C), where it is the lowest I-RMSD model among all 10,000. Cyan (right): the lowest I-RMSD model from entirely rigid-body docking. (A) SRP54 (PDB 2V3C). Flex-LZerD yielded a model with an I-RMSD of 6.9 Å, an L-RMSD of 5.5 Å, and an *f*_nat_ of 0.26, while rigid-body docking could at best sample a model with an I-RMSD of 14.8 Å, an L-RMSD of 21.5 Å, and an *f*_nat_ of 0.00. (B) 3′−5′ Exoribonuclease 1 (PDB 4QOZ). Flex-LZerD yielded a model with an I-RMSD of 4.6 Å, an L-RMSD of 8.7 Å, and an *f*_nat_ of 0.40, while rigid-body docking could at best sample a model with an I-RMSD of 11.3 Å, an L-RMSD of 10.2 Å, and an *f*_nat_ of 0.00. (C) Fab heavy/light chain (PDB 2R8S). The Flex-LZerD model shown has an I-RMSD of 3.8 Å, an L-RMSD of 21.7 Å, and an *f*_nat_ of 0.33, while rigid-body docking could at best sample a model with an I-RMSD of 6.0 Å, an L-RMSD of 36.4 Å, and an *f*_nat_ of 0.01. I-RMSD, interface RMSD; L-RMSD, ligand RMSD; PDB, Protein Data Bank; RMSD, root-mean-square deviation.

Highlighted in Figure 4A is signal recognition particle 54 kDa protein (SRP54), essential for cotranslational targeting of membrane proteins [33], bound to RNA as part of the targeting cycle. SRP54 must form interactions with the minor groove of the RNA to bind. For the blind ligand input, we used a structure of SRP54 that was determined separately (PDB: 3NDB). Domains were cut from the unbound structure by separating its NG domain from its M domain, removing their flexible linker [33] from Pro283 to Thr327. This binding consequently requires an overall conformational change of 13.8 Å. The rigid-body docking was only able to reach 14.8 Å I-RMSD. Flex-LZerD on the other hand was able to conform SRP54 to the minor groove with an I-RMSD of 5.8 Å using NG and M domain poses modeled to 2.3 Å I-RMSD and 6.2 Å I-RMSD, respectively. The progression from the input unbound SRP54 structure to this fitted model is illustrated in Supplemental Video S1. Both domains quickly reorient to match their separately docked counterparts, and steric complementarity with the receptor is quickly achieved as well. This target can be thought of as similar to the way Flex-LZerD was previously shown to model calmodulin [19], where two domains which do not tightly interact with each other wrap around a binding partner. As with calmodulin, these domains are joined by a linker which can transition between helical and coil secondary structures. This analogous example demonstrated that even when the receptor is not a protein, similar principles apply and flexible fitting can start from an open conformation model of a protein, could close the model in a way that accepts the protein’s binding partner, and could produce a model close to the native structure.

### 3.2 | Case study 2: 3′–5′ Exoribonuclease 1 (PDB 1ZBH/4QOZ)

Highlighted in Figure 4B is 3′–5′ exoribonuclease 1 (ERI1), which plays a part in the regulation of histone mRNA expression, binding directly to a stem-loop [66]. For the blind ligand input, we used a structure of ERI1 that was determined separately (PDB 1ZBH). Domains were cut from the unbound structure by separating its nuclease domain from its SAP domain, removing their flexible linker [66] from Lys112 to Tyr129. This binding requires an overall conformational change of 13.4 Å. The rigid-body docking was only able to reach 11.3 Å I-RMSD. Flex-LZerD on the other hand was able to conform ERI1 to the partner RNA with an I-RMSD of 4.63 Å using SAP and nuclease domain poses modeled to 3.7 Å I-RMSD and 2.6 Å I-RMSD, respectively. The progression from the input unbound ERI1 structure to this fitted model is illustrated in Supplemental Video S2. This target requires that rather than wrap around the RNA stem-loop, the protein should deform to form a long interface along the RNA. Flex-LZerD can thus handle flexible assemblies requiring the union of two previously disjoint binding sites into a single contiguous interface.

### 3.3 | Case study 3: Fab heavy/light chain (PDB 6APC/2R8S)

Figure 4C shows a fragment antigen-binding protein (Fab), an important part of both the animal immune system and the toolkits of many molecular biology research programs [67], bound to ribozyme RNA (PDB 2R8S). This target was especially challenging due to the fact that the Fab was synthetically designed to bind RNA, whereas the unbound structure was different, a natural protein-binding Fab (PDB 6APC). While Flex-LZerD was able to sample a correct binding pose, it was ultimately not able to select it. The complementarity-determining regions (CDRs) of antibodies that bind nucleic acids differ substantially from those that target other proteins [34]. For this particular target, the task was essentially to attempt to assemble a protein-binding Fab, with the unbound structure taken from PDB 6APC, with RNA. The bound structure from PDB 2R8S contains a synthetic Fab designed to target RNA with high specificity. Domains were cut from the unbound structure by extracting the variable domain of each of the heavy chain and the light chain. This binding consequently requires an overall conformational change of 10.7 Å.

Although this particular unbound Fab is a protein binder, its CDRs are not of substantially different size than those of the synthetic Fab. Thus, it was hoped that this unbound Fab may be at least sterically compatible with this ribozyme. The rigid-body docking was only able to sample as low as 6.0 Å I-RMSD. Flex-LZerD on the other hand was able to sample a Fab binding pose and conformation to the ribozyme with an I-RMSD of 3.8 Å. The progression from the input unbound Fab structure to this fitted model is illustrated in Supplemental Video S3. Here, the domains quickly deform to match their separately docked counterparts, and steric complementarity with the receptor is roughly achieved early on. However, while Flex-LZerD was able to sample the correct pose using what was essentially an incorrect, although appropriately sized, antibody, the current Flex-LZerD pipeline does not have a means of selecting such poses when sampled. Indeed, the output of the full pipeline was a wrong pose with an I-RMSD of 15.2 Å. This particular synthetic antibody uses highly specific hydrogen bonds to bind RNA [34], but the Flex-LZerD scoring function is not parameterized to directly consider hydrogen bonding interactions between nucleic acids and proteins. Thus, future development in this direction should incorporate a means of accounting for nucleic acid sequence specificity.

## 4 | CONCLUSION

In this work, we have demonstrated the docking by flexible fitting has the ability to handle many classes of protein–nucleic acid interactions. While state-of-the-art deep learning approaches like AlphaFold [11, 12] are highly developed for protein structure prediction, AlphaFold does not allow nucleic acids as queries. Protein–nucleic acid complex modeling is thus still relatively underdeveloped. The approach of Flex-LZerD, based in the more classical methods of domain docking and normal mode analysis, enables assembly of these molecules without needing to retool the entire pipeline. The current Flex-LZerD framework has inherent limits which inhibit modeling of certain other classes, including antibody–antigen interactions, domain size regimes where surface shape complementarity is less useful, or scenarios where nucleic acid specificity prediction is necessary [68]. The datasets used in this work rely on experimental structures of RNA where the interacting sequence region is essentially isolated from any longer strand. In practical blind use, it would be necessary to fix the nucleic acid sequence under consideration. However, clear directions are available to surmount all these challenges. Cases of very small domains and long loop rearrangements can be modeled using techniques from intrinsically-disordered region docking [52] or deep learning [11]. Cases where no bound nucleic acid structure is available, or where complementarity cannot be relied upon, could be approached with sequence specificity prediction methods [68]. We therefore anticipate improvements along these lines as part of future developments.

## Supplementary Material

Supplementary Information

Supplementary Video 1

Supplementary video2

Supplementary video 3

## Figures and Tables

**TABLE 1:** The expanded benchmark set of protein-nucleic acid targets

Ligand protein name	Native complex PDB	Total complex #residues	Unbound ligand PDB	Ligand conformational difference (Cα RMSD, Å)

Signal recognition particle 54 kDa protein	2V3C (AM:C)	1172	3NDB (C)	13.8
DNA polymerase IV	2W9B (CE:A)	745	2RDI (A)	18.2
*DNA polymerase IV	2IMW (ST:P)	379	3FDS (A)	16.3
*DNA polymerase beta	6NKZ (DPT:A)	366	1BPD (A)	11.9
3’-5’ exoribonuclease 1	4QOZ (AC:B)	674	1ZBH (A)	13.4
Ribonuclease E	6G63 (B:AG)	1997	5F6C (AB)	11.5
Histone H3.3	6NQA (ABCDFGHIJKL:E)	1456	5KDM (A)	10.1
Transcription factor p65	2I9T (BCD:A)	621	1NFI (A)	10.4
*Transcription initiation factor IIB	1C9B (BCD:A)	421	5WH1 (A)	12.2
Nuclear factor of activated T-cells, cytoplasmic 2	1P7H (ABM:L)	1204	2AS5(N)	16.5
*Transcriptional activator Myb	1H89 (ABDE:C)	337	1GV2 (A)	7.1
Elongation factorTu 2	1OB2 (B:A)	470	4ZV4 (A)	11.8
*Elongation factorTu	1TTT (D:A)	482	1AIP (A)	11.4
Fab heavy/light chain	2R8S (R:HL)	592	6APC (HL)	10.7
*RP-A 70 kDa DNA-binding subunit	1JMC (B:A)	256	1FGU (A)	8.3
*Antiviral innate immune response receptor RIG-I	7JL1 (XY:A)	750	4ON9 (A)	13.2
*Phenylalanine-tRNA ligase, mitochondrial	3TUP (T:A)	491	5MGU (A)	18.7

Asterisks (*) indicate targets also present in the original Flex-LZerD dataset. The native protein ligands of the two polymerase IV entries, 2W9B and 2IMW, have 100% sequence identity, but their bound DNA molecules have 87% sequence identity in terms of standard bases and additionally contain different nonstandard bases, with an RMSD of 5.4 A. The native protein ligands of two elongation factor Tu entries, 1OB2 and 1TTT, have 74% sequence identity, and their bound RNA molecules have 98% sequence identity and additionally contain different nonstandard bases, with an RMSD of 2.0 A. The remaining targets have less than 25% sequence identity between each other. PDB, Protein Data Bank; RMSD, root-mean-square deviation.

**TABLE 2:** Docking performance of individual targets

Ligand PDB	Domain level				Complex level (I-RMSD, Å)				
	CAPRI-acceptable hits	Best *f*_nat_ in top 10	Best I-RMSD in top 10 (Å)	Best L-RMSD in top 10 (Å)	Flex-fitting-to-native	Rigid-body LZerD	Top-scored	Best in top 10	Best in all

3NDB	8/1	0.22/0.10	1.58/4.90	3.40/10.95	4.05	(14.84)	6.88	6.68	5.75
2RDI	7/5	0.30/0.42	2.45/1.33	5.17/3.01	3.86	(14.10)	4.97	4.97	4.10
*3FDS	1/1	0.59/0.43	1.99/4.92	4.39/5.68	5.54	(15.33)	11.04	11.04	9.58
*1BPD	5/6	0.50/0.29	2.69/2.94	7.46/5.18	2.04	(11.58)	4.54	4.48	4.48
1ZBH	13/2	0.34/0.27	0.91/2.62	3.03/9.23	1.58	(11.25)	4.63	4.63	3.01
5F6C	12/0	0.25/0.29	2.07/6.66	11.40/30.46	1.97	2.32	3.98	3.98	3.51
5KDM	0/0	0.07/0.04	0.62/8.51	3.6/23.33	1.45	(12.77)	(11.42)	(11.41)	(8.77)
1NFI	2/9	0.24/0.43	2.24/0.89	6.95/2.51	4.34	(7.83)	4.57	4.57	4.11
*5WH1	8/3	0.16/0.11	2.20/0.12	5.55/1.98	2.45	(8.73)	4.08	4.08	4.08
2AS5	11/9	0.35/0.23	1.49/1.42	6.66/4.39	2.78	(10.45)	4.61	4.61	2.85
*1GV2	2/3	0.81/0.76	1.92/0.92	4.14/1.90	2.08	(6.13)	3.39	3.31	3.03
4ZV4	0/16	0.10/0.51	4.96/1.60	12.16/4.02	3.59	(10.87)	(10.21)	(10.21)	(9.47)
*1AIP	1/4	0.89/0.77	3.74/1.86	15.98/4.52	2.75	(8.30)	4.09	4.09	4.08
6APC	1/6	0.25/0.16	2.88/3.24	6.99/6.15	1.91	(5.99)	(15.24)	(14.77)	3.82
*1FGU	7/3	0.70/0.45	1.71/3.86	3.74/7.57	1.62	(9.56)	5.91	5.43	5.40
*4ON9	6/2	0.69/0.17	2.15/5.78	5.18/8.98	2.51	(11.22)	4.69	4.68	4.59
*5MGU	0/0	0.41/0.11	4.99/6.45	19.77/16.99	4.99	(14.22)	8.26	6.92	6.92

Targets are identified by their ligand PDB IDs. “/” indicates that the two numbers given are for the first and second domains, respectively. Domain hits, the number of CAPRI-acceptable quality domains within the top 100 by the combined scoring function. Numbers given in parentheses indicate models which did not meet the CAPRI-acceptable quality criteria. Flexible-fitting-to-native results are for the single output model generated when performing flexible fitting of the unbound structure directly to the bound native structure. Rigid-body LZerD results showthe best I-RMSD in the entire rigid-body LZerD pipeline output set using the unbound ligand conformation, typically tens of thousands of models. Asterisks (*) indicate targets also present in the original Flex-LZerD dataset. I-RMSD, interface RMSD; L-RMSD, ligand RMSD; PDB, Protein Data Bank; RMSD, root-mean-square deviation.

## Data Availability

The data that support the findings of this study are openly available in Zenodo at https://doi.org/10.5281/zenodo.7412584, reference number [65].

## Significance Statement

Computational modeling of protein–nucleic acid complex helps mechanistic understanding of the function of DNA/RNA binding proteins. Although a protein often undergoes significant conformational changes upon docking with nucleic acids, most of the existing docking methods do not consider such large-scale flexibility. Here, we show that our method, Flex-LZerD, can model complexes with extremely large conformational changes.

## Abbreviations:

RMSD: root-mean-square deviation
I-RMSD: interface RMSD
L-RMSD: ligand RMSD
fnat: fraction of native contacts
RTB: rotations and translations of blocks
BSC: binding site consensus
