# Supplementary material for: Modeling protein–nucleic acid complexes with extremely large conformational changes using Flex-LZerD
Source: Proteomics. Author manuscript; Available in PMC 2024 Sep 1. (PMC10448949; doi:10.1002/pmic.202200322)
Supplement: Supplementary Information [file NIHMS1923186-supplement-Supplementary_Information.pdf]

Supplemental materials for
Modeling Protein-Nucleic Acid Complexes with Extremely Large Conformational Changes using Flex-LZerD

Charles Christoffer[1] and Daisuke Kihara[1,2,*]

[1] Department of Computer Science, Purdue University, West Lafayette, Indiana, 47907, USA
[2] Department of Biological Sciences, Purdue University, West Lafayette, Indiana, 47907, USA

* Corresponding author.
E-mail: dkihara@purdue.edu

Supplemental Video S1. (in a separate file; in MP4 format). Fitting progression of signal recognition particle 54 kDa protein (SRP54) docking to RNA. Gray: the receptor structure from 2V3C. Magenta: the docked SRP54 domain models used to guide the fitting. Cyan: the unbound SRP54 ligand from PDB 3NDB being flexibly docked. The final frame model has an I-RMSD of 6.9 Å, an L-RMSD of 5.5 Å, and an f_{nat} of 0.26.

Supplemental Video S2. (in a separate file; in MP4 format). Fitting progression of 3'-5' exoribonuclease 1 (ERI1) docking to RNA. Gray: the receptor structure from PDB 4QOZ. Magenta: the docked ERI1 domain models used to guide the fitting. Cyan: the unbound ERI1 ligand from PDB 1ZBH being flexibly docked. The final frame model has an I-RMSD of 4.6 Å, an L-RMSD of 8.7 Å, and an f_{nat} of 0.40.

Supplemental Video S3. (in a separate file; in MP4 format). Fitting progression of a Fab region docking to RNA, the best (by I-RMSD) sampled by Flex-LZerD. Gray: the receptor structure from PDB 2R8S. Magenta: the docked Fab domain models used to guide the fitting. Cyan: the unbound Fab ligand from PDB 6APC being flexibly docked. The final frame model has an I-RMSD of 3.8 Å, an L-RMSD of 21.7 Å, and an f_{nat} of 0.33.
